# Urine Metanephrine Concentration Can Early and Accurately Predict Etiology of Acute Respiratory Failure in Critically Ill Patients with Subarachnoid Hemorrhage: A Prospective Single-Center Pilot Study

**DOI:** 10.3390/jcm15041557

**Published:** 2026-02-16

**Authors:** Mateusz N. Zachura, Natalia Kopcińska, Michał P. Pluta, Mateusz Gołdyn, Bartosz Blada, Dominika Krupnik, Magdalena Kwiatkowska, Łukasz J. Krzych

**Affiliations:** 1 Department of Anesthesiology and Intensive Care, Upper-Silesian Medical Center, 40-635 Katowice, Poland; michal.pluta@sum.edu.pl (M.P.P.);; 2 Department of Acute Medicine, Faculty of Medical Science in Zabrze, Medical University of Silesia, 41-800 Zabrze, Poland; 3 Students’ Scientific Society “#Intensywna_Po_Godzinach”, Department of Acute Medicine, Medical University of Silesia, 41-800 Zabrze, Poland; mateusgoldyn@gmail.com (M.G.); mika16.dk@gmail.com (D.K.);

**Keywords:** subarachnoid hemorrhage, acute respiratory failure, metanephrine, interleukin-6

## Abstract

**Background:** Subarachnoid hemorrhage (SAH) can cause remote organ failure through complex systemic reactions. Acute respiratory failure (ARF) in the course of SAH may have a diverse etiology, including cardiogenic origin. The aim of the study was to evaluate the utility of urine metanephrine measurement in identifying the ARF phenotype in patients with SAH. **Methods:** A prospective single-center study was conducted between January 2022 and February 2023. The study included consecutive adult patients admitted to the Intensive Care Unit (ICU) within 24 h of SAH diagnosis and requiring mechanical ventilation due to ARF within the first 48 h of stay. Demographic and clinical data were collected. Metanephrine (MET) was determined in 24-h urine collection. The inflammatory profile was assessed by measuring serum levels of interleukin-6 (IL-6), CRP, and PCT. Cardiogenic ARF phenotype was diagnosed when concomitant elevation of hsTpI, CK-MB, and NT-proBNP was observed upon admission. **Results:** The study group consisted of 18 patients. The cardiogenic etiology group (*n* = 4) was characterized by higher MET concentrations (249 vs. 63.5 ng/mL; *p* = 0.007) and a lower oxygenation index (190 vs. 296 mmHg; *p* < 0.05) on admission. In the non-cardiogenic etiology group (*n* = 14), higher levels of IL-6 were found (34 vs. 8.3 pg/mL; *p* = 0.013). MET significantly correlated with the oxygenation index (R = −1.0; *p* < 0.001) on day 1 and with lactate levels on days 2 and 3 of stay (R = 1.0; *p* < 0.001). Baseline MET concentration accurately predicted the ARF phenotype (AUC 0.93; 95% CI 0.786–1.000, *p* = 0.008). **Conclusions:** Urine metanephrine levels show potential in differentiating the etiology of ARF and correlate with severity markers in critically ill SAH patients at an early stage. These preliminary results highlight the importance of a targeted approach to ARF diagnostics after SAH, which could support appropriate therapeutic decisions, although further validation in larger cohorts is required.

## 1. Introduction

Although subarachnoid hemorrhage (SAH) is a pathology primarily located in the central nervous system, it often leads to systemic complications [1,2]. Mortality associated with SAH is substantial, ranging from 35% to 45%, with a significant proportion of deaths occurring within the first 48 h after the hemorrhage [3]. Among patients surviving the acute phase, up to 70% experience unfavorable neurological and functional outcomes, resulting not only from the primary neurological injury but also from secondary complications [4].

It has been demonstrated that SAH activates complex systemic reactions in which so-called neuro-organ axes, including the brain–heart and brain–lung axes, play a pivotal role [5,6]. Increased sympathetic activity induced by SAH leads to direct lung injury through systemic inflammatory response, pulmonary microcirculatory disturbances, and increased permeability of the alveolar–capillary barrier. Concurrently, adverse hemodynamic sequelae occur in a subset of patients. Elevated left ventricular filling pressure caused by severe hypertension, neurogenic cardiomyopathy, or heart failure may additionally damage pulmonary capillaries via increased hydrostatic pressure [7,8,9]. While laboratory assessment of troponin, CK-MB, and NT-proBNP levels, hemodynamic monitoring via Swan–Ganz catheter, and echocardiography are well-established in the diagnosis of cardiac injury, the determination of biomarkers of sympathetic activation (i.e., catecholamines and their metabolites) is not routine practice.

The aim of this study was to evaluate the utility of urine metanephrine measurement in the early identification of the acute respiratory failure (ARF) phenotype in patients with SAH admitted to the ICU. The analysis focused on differentiating between two main phenotypes of this dysfunction, cardiogenic and non-cardiogenic, in order to determine the most probable mechanism responsible for the development of hypoxemia in the studied population of critically ill patients.

## 2. Materials and Methods

### 2.1. Study Design

This prospective 7-day study was conducted at the Intensive Care Unit (ICU) of a multidisciplinary university hospital between January 2022 and February 2023. The study received approval from the Bioethics Committee at the Medical University of Silesia in Katowice (PCN/0022/KB/8/21). The research protocol was also registered in the online ClinicalTrials.gov database (NCT05408988). The research project was funded by the Ministry of Education and Science under the program “Studenckie Koła Naukowe Tworzą Innowacje” (SKN/SP/534909/2022).

### 2.2. Patients

The study included adult patients with SAH confirmed by head computed tomography (CT), who were admitted to the ICU within the first 24 h of hospital presentation and required at least 7 days of ICU treatment. Inclusion criteria also required endotracheal intubation and invasive mechanical ventilation due to ARF defined as an oxygenation ratio (OI) (PaO_2_/FiO_2_) below 300 mmHg within the first 48 h post-event. Patients meeting any of the following criteria were excluded from the study:

- History of severe lung disease (e.g., COPD, bronchial asthma, pulmonary fibrosis, pulmonary hypertension);
- History of severe heart disease (e.g., cardiomyopathies, severe valvular defects, status post cardiac surgery, status post coronary revascularization);
- Death within the first 24 h of ICU admission;
- Need for any extracorporeal support techniques (e.g., ECMO, CRRT);
- Diagnosed pulmonary embolism on admission.

Qualified patients underwent a seven-day clinical observation starting from the moment of ICU admission.

### 2.3. Clinical and Laboratory Data

Using electronic medical records available in the AMMS system (Asseco, Gdańsk, Poland), the following demographic and clinical data were collected: sex, age, SAH etiology, aneurysm location, neurological status on hospital admission assessed by the Glasgow Coma Scale (GCS), symptom severity by the Hunt–Hess scale, hemorrhage severity on CT by the Fisher scale, modified WFNS scale, APACHE II scale, SAPS II scale, history of comorbidities and their treatment, substance use, and the method and efficacy of SAH treatment. Mechanical ventilation parameters, correlated with arterial blood gas results, were recorded twice daily.

All patients were routinely catheterized during their stay. A 24-h urine collection was utilized for study purposes. To maintain urine pH < 3 and prevent the degradation of metanephrine fractions, 10 mL of 20% hydrochloric acid (HCl) was added to the urine bag as a stabilizer for each collection. Urine samples were collected every 12 h (i.e., two samples per day). Samples were subsequently frozen at −70 °C, and metanephrine levels were determined after the observation period of the last recruited patient was completed. Measurements were performed using a competitive enzyme-linked immunosorbent assay (ELISA) according to the manufacturer’s instructions for the Demeditec Metanephrine Urine ELISA DEE8400 kit (Demeditec Diagnostics GmbH, Cologne, Germany). The analysis was intentionally limited to metanephrine measurements, excluding normetanephrine assessment. This decision was dictated by the need to eliminate the confounding factor of exogenous catecholamine administration (all patients received norepinephrine infusion to maintain cerebral perfusion pressure; no patient received epinephrine infusion).

During the ICU stay, all patients had an arterial catheter and venous access. Arterial blood gas analysis was performed twice daily using the RAPIDPoint^®^ 500e critical parameter analyzer (Siemens Healthineers, Erlangen, Germany). Once daily, the following markers were measured: IL-6, IL-1β, C-reactive protein (CRP), procalcitonin (PCT), high-sensitivity troponin I (hs-TnI), total creatine kinase (CPK) and its CK-MB isoenzyme, and B-type natriuretic peptide (NT-proBNP), according to procedures available at the central hospital laboratory. Venous blood samples were drawn into dedicated tubes (BD Vacutainer, Becton Dickinson, Swindon, UK) and immediately transported to the hospital laboratory.

Patients with acute hypoxemic respiratory failure (ARF), defined as an oxygenation ratio (PaO_2_/FiO_2_) < 300 mmHg, underwent screening for respiratory failure etiology (*n* = 18). In this group, ARF etiology was evaluated based on the myocardial injury biomarker profile. Significant biochemical features of cardiomyocyte injury (i.e., concomitant elevation of laboratory levels of hs-TnI, CK-MB, and NT-proBNP) were found in 4 patients, which was considered a cardiogenic etiology of the respiratory failure mechanism. Patients who did not show elevated myocardial injury markers (*n* = 14) and met the criteria of the new 2024 global definition of acute respiratory distress syndrome (ARDS) [10] were classified into the non-cardiogenic lung injury phenotype group. This classification aimed to identify distinct pathophysiological mechanisms of ARF in the course of SAH.

Diagnostic imaging followed a standardized protocol. Upon admission, a baseline chest radiograph (CXR) was obtained in all patients to confirm catheter placement and exclude pneumothorax. For the subsequent diagnosis of ARF and daily monitoring, bedside Lung Ultrasound (LUS) was utilized as the primary imaging modality, in accordance with the 2024 global definition of ARDS. To minimize radiation exposure and avoid the risks associated with intrahospital transport of critically ill SAH patients, follow-up CXR and chest HRCT were not performed routinely. These modalities were reserved for specific clinical indications, particularly to differentiate between atelectasis and infectious consolidation in patients with suspected ventilator-associated pneumonia.

### 2.4. Statistical Analysis

Statistical analysis was performed using MedCalc 18 software (MedCalc Software bvba, Ostend, Belgium). Qualitative variables are presented as absolute values and percentages. Quantitative variables are presented as medians (Me) and interquartile ranges (IQR, 25–75 pc) due to their non-normal distribution. The non-parametric Mann–Whitney U test was used to compare quantitative variables between two groups. Spearman’s rank correlation analysis (R) was used to assess the strength and direction of relationships between studied parameters. The diagnostic and predictive value of biomarkers was evaluated using ROC curve analysis. The area under the curve (AUROC) with a 95% confidence interval (95% CI) was calculated to determine the discriminatory ability of the tests. In all analyses, *p* < 0.05 was considered statistically significant.

## 3. Results

**Figure 1:**
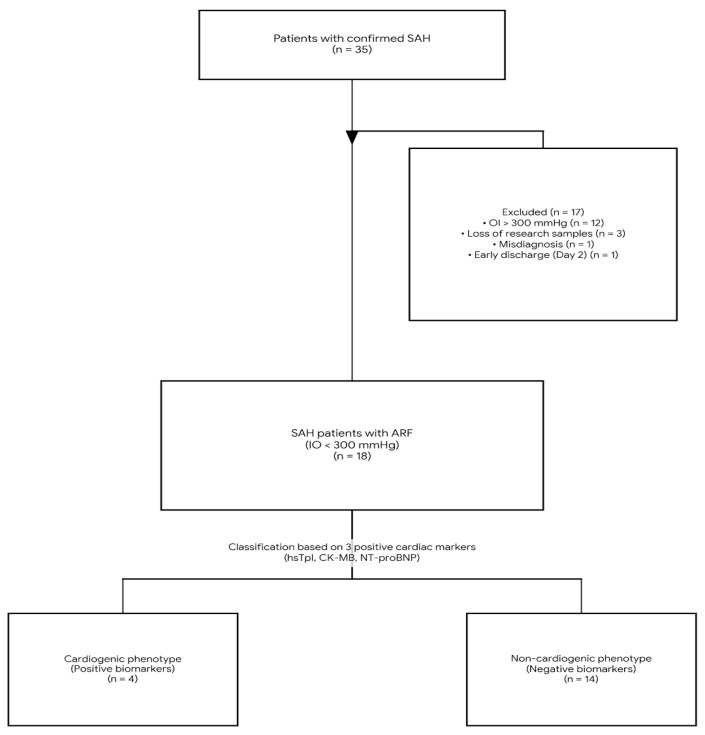
CONSORT diagram.

Thirty-five patients were initially enrolled in the study; however, 17 patients were excluded (1 patient due to misdiagnosis, 3 patients due to loss of research samples, 1 patient due to discharge to the neurology department on the second day post-SAH, and 12 patients presented with an OI > 300 mmHg within the first 48 h of admission) (Figure 1). The final analysis included 18 patients, including 11 women (61%). The median age was 57 (IQR 44–65) years. The in-hospital mortality rate in the study group was 50% (9/18 patients). The primary cause of death was irreversible neurological injury in 8 cases (3 in the cardiogenic group and 5 in the non-cardiogenic group). One patient in the non-cardiogenic group died due to septic shock complicating ventilator-associated pneumonia. Importantly, despite the presence of myocardial dysfunction in the cardiogenic group, no deaths were directly attributed to primary cardiac events. Baseline demographic and clinical data are presented in Table 1.

Hemodynamic management was standardized across both groups, targeting a Mean Arterial Pressure (MAP) > 80 mmHg (or >100 mmHg in cases of vasospasm) to ensure adequate cerebral perfusion. Consequently, all patients (100%) received nimodipine for vasospasm prophylaxis and norepinephrine as the first-line vasopressor. To manage refractory hypotension, second-line therapy with arginine vasopressin was required in 3 patients in the cardiogenic group (all of whom were non-survivors) and 5 patients in the non-cardiogenic group. Conversely, following hemodynamic stabilization and weaning from vasopressors, continuous urapidil infusion was initiated to control hypertension in 1 patient in the cardiogenic group and 6 patients in the non-cardiogenic group. Due to this goal-directed therapy, the achieved MAP levels remained within the therapeutic target range and were comparable between groups throughout the observation period.

**Figure 2:**
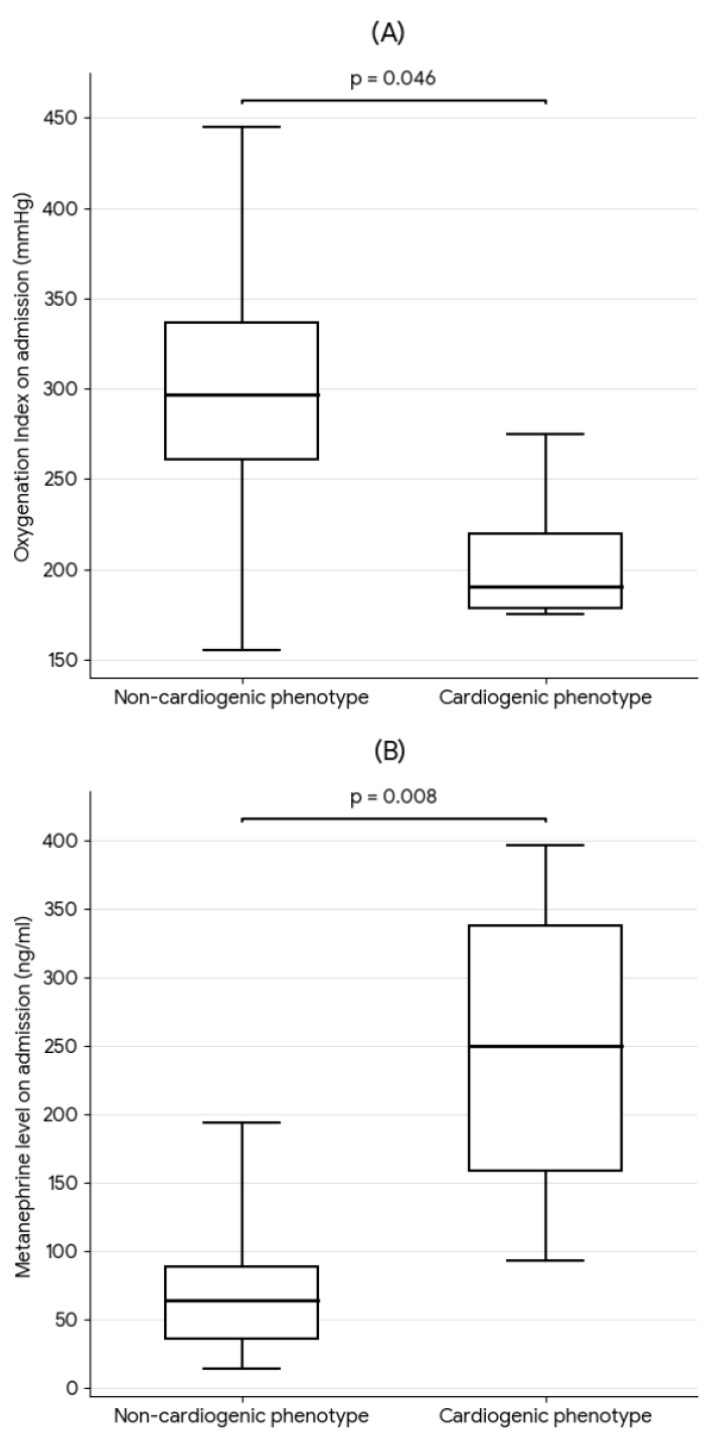

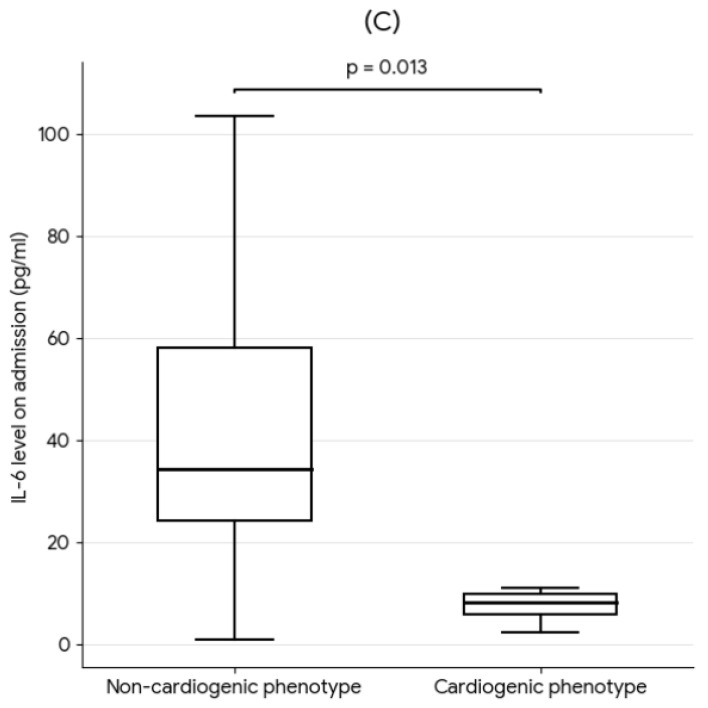
Oxygenation index (**A**), metanephrine (**B**), and interleukin-6 (**C**) levels on admission in cardiogenic and non-cardiogenic ARF phenotype groups. The horizontal line indicates the median (Me). The box represents the interquartile range (IQR, 25th–75th percentiles). Whiskers indicate the minimum and maximum values.

Statistically significant differences in respiratory and biochemical parameters were observed between groups with distinct ARF phenotypes. Patients with a cardiogenic mechanism of lung injury were characterized by a significantly lower oxygenation ratio on admission (median 190 vs. 296 mmHg; *p* < 0.05). Moreover, nearly fourfold higher metanephrine concentrations in 24-h urine collection were found in this group on admission (median 249 vs. 63.5 ng/mL; *p* = 0.007). In the group with the non-cardiogenic ARF phenotype, significantly higher IL-6 levels were recorded (median 34 vs. 8.3 pg/mL; *p* = 0.013) (Figure 2). A comparative analysis of groups with different ARF etiologies is presented in Table 2.

**Figure 3:**
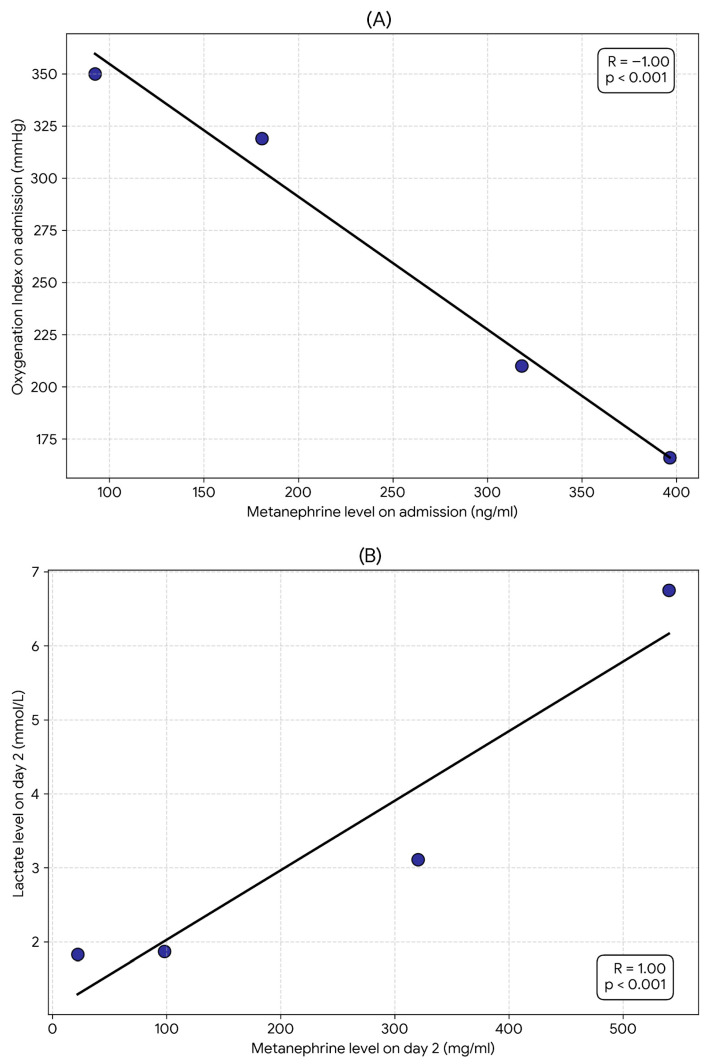

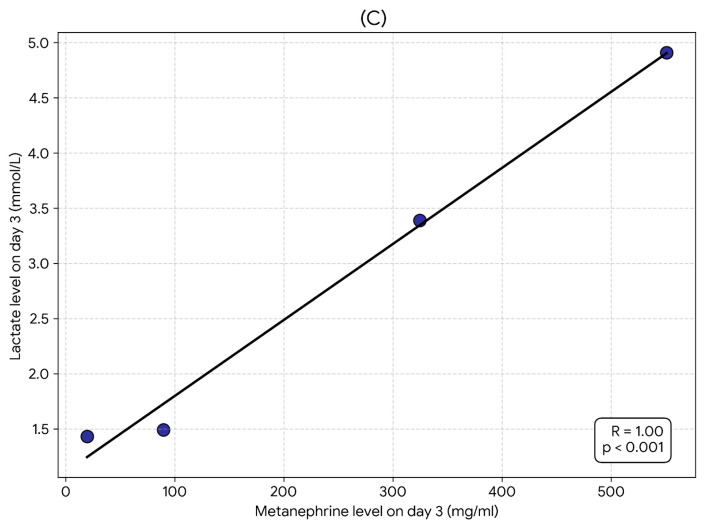
Correlation in the cardiogenic phenotype group between Met and OI on admission (**A**), Met and Lac on day 2 (**B**), Met and Lac on day 3 (**C**).

In the cardiogenic phenotype group, a strong, negative, and statistically significant correlation was demonstrated between metanephrine levels and the oxygenation ratio: on day 1 of observation, the correlation coefficient was R = −1.0 (*p* < 0.001). A strong, positive, and statistically significant correlation was also observed between metanephrine levels and serum lactate levels; on days 2 and 3 of treatment, the correlation coefficients were R = 1.0 (*p* < 0.001) (Figure 3).

**Figure 4:**
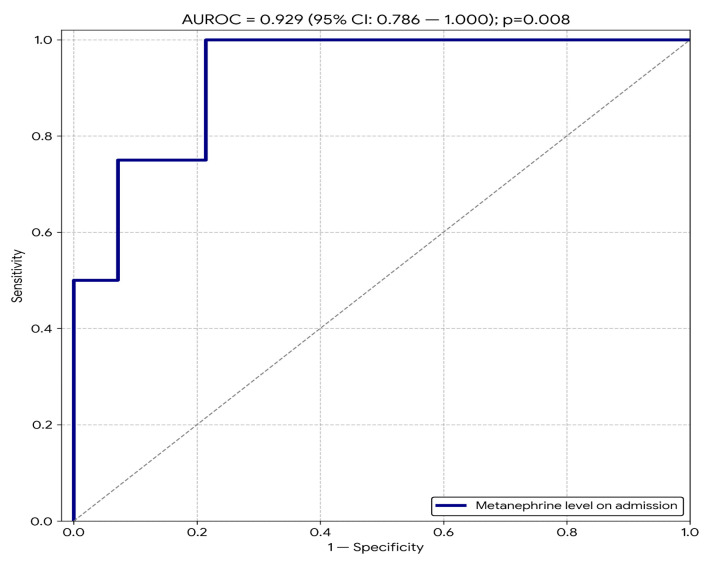
Diagnostic accuracy of metanephrine on day 1 of ICU stay in predicting the ARF phenotype.

The level of the metanephrine on the first day of hospitalization demonstrated a very high discriminatory ability in identifying the mechanism of acute respiratory failure (AUROC = 0.929; 95% CI 0.786–1.000; *p* = 0.008) (Figure 4).

**Figure 5:**
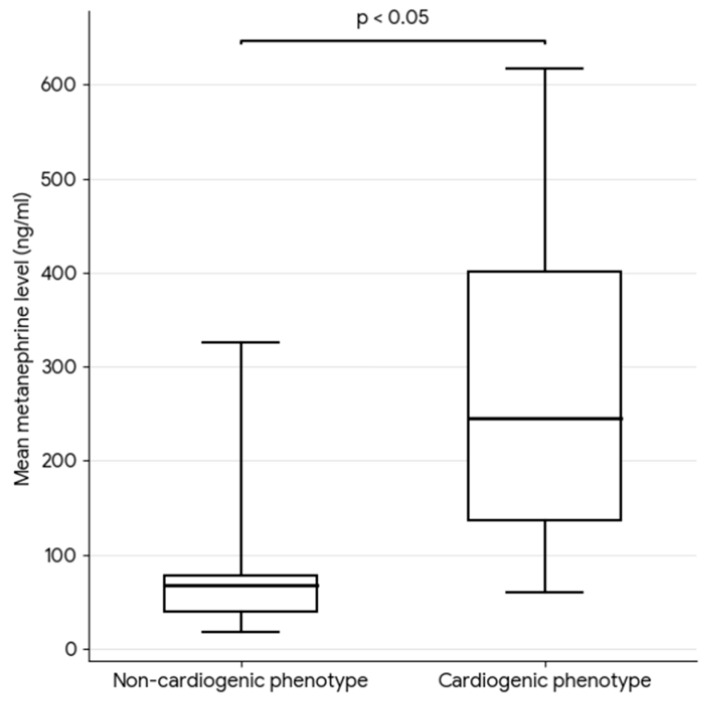
Mean metanephrine level during 7 days of ICU hospitalization in cardiogenic and non-cardiogenic ARF phenotype groups. The horizontal line indicates the median (Me). The box represents the interquartile range (IQR, 25th–75th percentiles). Whiskers indicate the minimum and maximum values.

Although the prognostic value of metanephrine measurement on day 1 of the stay regarding in-hospital mortality was not confirmed (AUROC = 0.64; *p* > 0.05), patients in the cardiogenic ARF phenotype group were characterized by significantly higher mean urine metanephrine concentrations during the first 7 days of ICU hospitalization (292.16 vs. 79.87 ng/mL, *p* < 0.05) (Figure 5).

## 4. Discussion

The aim of this study was to evaluate the utility of urine metanephrine measurement in the early identification of the ARF phenotype in SAH patients admitted to the ICU. Statistically significant differences were demonstrated in OI values, metanephrine levels, and IL-6 levels on the first day after SAH between groups with different etiologies of lung injury. Furthermore, metanephrine levels were found to correlate positively with ARF severity. Importantly, metanephrine levels did not predict in-hospital mortality. The lack of significant association with mortality is likely attributable to the limited sample size. Furthermore, survival in SAH is predominantly determined by the severity of the primary brain injury and delayed neurological sequelae, rather than acute, often reversible, cardiorespiratory complications reflected by early metanephrine peaks.

The obtained results are consistent with prior observations indicating that SAH initiates a complex cascade of systemic reactions, including both sympathoadrenal system activation and an inflammatory response. Classical studies have demonstrated that a significant increase in the urinary excretion of catecholamines and their metabolites, including metanephrine and normetanephrine, occurs within the first few days after SAH, reflecting excessive activation of the hypothalamus–brainstem–adrenal medulla axis [11]. It has also been shown that plasma epinephrine and norepinephrine levels increase proportionally to hemorrhage severity, leading to elevated blood pressure, tachycardia, and increased anaerobic metabolism [12]. This phenomenon is termed a “catecholamine storm,” which can induce toxic effects on the myocardium and perfusion disturbances in the pulmonary microcirculation. This was confirmed in the group of patients with a cardiogenic component, where a significant positive correlation was observed between metanephrine levels and lactate levels, indicating transient tissue hypoperfusion.

The association between excessive catecholamine release and the occurrence of extracerebral complications of SAH, including ARF, has been confirmed in clinical and experimental studies [9]. Excessive sympathetic activation has been shown to lead to increased pulmonary capillary pressure, increased alveolar–capillary barrier permeability, and transient pulmonary vasoconstriction, which clinically manifests as the development of neurogenic pulmonary edema [9]. Our results, indicating a significant correlation between metanephrine levels and a decrease in the OI, confirm that the intensity of neurogenic sympathetic activation may play a key role in the pathogenesis of early respiratory disturbances following SAH.

The relationship between catecholamine release and myocardial injury in the course of SAH has been confirmed in clinical studies [13,14]. It has been demonstrated that elevated myocardial injury markers in these patients are associated with transient left ventricular systolic dysfunction and the occurrence of regional wall motion abnormalities, defined as neurogenic cardiomyopathy. This mechanism is directly related to the toxic effect of catecholamines, leading to excessive calcium ion influx into cardiomyocytes, oxidative stress, and cellular edema [15]. In our study, patients with positive myocardial injury markers presented with significantly higher metanephrine values, and ROC curve analysis indicated a very high diagnostic ability of this parameter in differentiating the ARF phenotype. These results are consistent with the concept that in a subset of SAH patients, secondary heart failure plays a dominant role in the development of respiratory failure, whereas in others, neurogenic and inflammatory mechanisms prevail. However, it must be emphasized that such a high diagnostic value, obtained in a limited sample size (*n* = 18), requires cautious interpretation and validation in multicenter studies to exclude the risk of overfitting. Nevertheless, this result indicates the potential of metanephrine as an early biomarker for cardiopulmonary risk stratification.

A particularly interesting and novel observation is the distinct inflammatory profile in the study groups. In contrast to reports suggesting a simple synergism between adrenergic and inflammatory activation [16,17,18], in our study, higher IL-6 concentrations were observed in the group of patients with a non-cardiogenic mechanism of respiratory failure (*p* = 0.013), with concomitant lower procalcitonin levels. This suggests the existence of a pathomechanism of lung injury in the course of SAH associated with a systemic inflammatory response or early infection, leading to increased pulmonary capillary permeability without a cardiogenic component.

The obtained results have significant clinical implications for the management of SAH patients. The demonstrated relationship between metanephrine levels and worsening oxygenation suggests that measurement of this metabolite may serve as an early, indirect indicator of excessive sympathetic activation and the risk of developing extracerebral SAH complications. Incorporating metanephrine assessment into standard monitoring, alongside blood gas parameters and cardiac markers, could facilitate the early differentiation of the respiratory failure phenotype—cardiogenic or non-cardiogenic.

Based on our pilot data, we propose a conceptual framework to address the therapeutic conflict between lung protection (fluid restriction) and cerebral perfusion (euvolemia). We suggest that identifying the ARF phenotype via echocardiography and urine metanephrine could guide management. Specifically, a cardiogenic profile (high metanephrine, elevated myocardial injury markers) supports fluid restriction, whereas a non-cardiogenic profile (low metanephrine, normal myocardial injury markers) favors euvolemia to prevent DCI. These preliminary observations are hypothesis-generating and warrant validation in larger cohorts. Such an approach would align with the concept of personalized medicine in SAH patients, aimed at limiting the systemic sequelae of primary brain injury.

### Study Limitation

Despite the significant observations obtained, this study has several limitations that must be considered when interpreting the results. First and foremost, the sample size was limited due to single-center recruitment over a one-year period (in accordance with the project’s funding constraints). It should be noted that the annual incidence of SAH is approximately 6–9 cases per 100,000 persons, indicating that it is a relatively infrequent condition in the general population; thus, a significantly larger cohort could not be expected within a 12-month observation period.

Second, although all patients underwent transthoracic echocardiography upon admission, this was performed as a focused point-of-care ultrasound (POCUS) by the attending intensivists, rather than a comprehensive echocardiographic examination. Consequently, phenotype classification relied primarily on the biomarker panel. We acknowledge that the absence of invasive hemodynamic monitoring (e.g., pulmonary artery catheterization) or comprehensive echocardiography limits the precision of differentiating complex, mixed-etiology cases. Furthermore, although patients with a documented history of severe cardiac disease were strictly excluded from the study, the lack of pre-admission echocardiographic data makes it impossible to definitively rule out the presence of undiagnosed, asymptomatic pre-existing myocardial dysfunction in some individuals. Consequently, while the clinical presentation strongly suggested acute neurogenic stunned myocardium, the baseline cardiac status could not be fully verified.

Another limitation worth mentioning is the stratification of patients into clinical groups based on available biochemical, gasometric, and radiological criteria, which may complicate the unequivocal classification of the respiratory failure type and lead to a potential overlap between cardiogenic and non-cardiogenic mechanisms. A further limitation is the lack of long-term follow-up and assessment of late neurological (mRS, CPC) or functional (Barthel Index) outcomes among survivors discharged from the hospital.

Finally, the substantial exclusion rate introduces a potential selection bias. The majority of excluded patients (12 out of 17) were removed due to an OI > 300 mmHg, meaning they did not exhibit significant respiratory failure. Therefore, our study effectively selected a cohort with more severe clinical presentations. While this was necessary to test the biomarker’s utility in distinguishing ARF phenotypes, it implies that our findings, particularly the correlations with severity markers, may not be generalizable to the broader SAH population with mild or no respiratory complications.

Although we reported nearly perfect correlations (R = ±1.0), which were statistically significant, they should be interpreted with caution. The obtained results should be considered preliminary and require confirmation in larger, prospective multicenter studies utilizing comprehensive organ assessment, aimed at validating metanephrine as a biomarker differentiating lung injury phenotypes in this patient population. Therefore, the presented results were treated as a pilot study for future research projects with broader funding scopes.

## 5. Conclusions

Assessment of urine metanephrine concentration in critically ill patients on the first day following SAH accurately predicts the etiology of acute respiratory failure. Our results highlight the importance of a targeted approach to ARF diagnostics after SAH, which may significantly influence appropriate therapeutic decisions. However, the findings require verification in further studies involving larger patient cohorts.

## Figures and Tables

**Table 1:** Baseline demographic and clinical characteristics.

Variable	Total *N* = 18	Non-Cardiogenic Phenotype *N* = 14	Cardiogenic Phenotype *N* = 4
Fisher scale, *n* (%):			
1	4 (22%)	3 (21%)	1 (25%)
2	1 (6%)	1 (7%)	0 (0%)
3	1 (6%)	1 (7%)	0 (0%)
4	12 (66%)	9 (65%)	3 (75%)
Hunt–Hess scale, *n* (%):			
1	3 (16%)	3 (21%)	0 (0%)
2	1 (6%)	1 (7%)	0 (0%)
3	1 (6%)	1 (7%)	0 (0%)
4	8 (44%)	6 (44%)	2 (50%)
5	5 (28%)	3 (21%)	2 (50%)
WFNS scale, *n* (%):			
1	3 (16%)	2 (14%)	1 (25%)
2	1 (6%)	1 (7%)	0 (0%)
3	1 (6%)	1 (7%)	0 (0%)
4	7 (39%)	5 (36%)	2 (50%)
5	6 (33%)	5 (36%)	1 (25%)
Cause of SAH, *n* (%):			
Ruptured aneurysm	10 (55%)	8 (58%)	2 (50%)
Arteriovenous malformation	1 (6%)	1 (7%)	0 (0%)
Trauma	2 (11%)	1 (7%)	1 (25%)
Vertebral artery dissection	3 (17%)	2 (14%)	1 (25%)
Iatrogenic	2 (11%)	2 (14%)	0 (0%)
Location of aneurysm, *n*(%):			
Anterior communicating artery	4 (22%)	4 (28%)	0 (0%)
Middle cerebral artery	8 (44%)	8 (58%)	0 (0%)
Internal carotid artery	4 (22%)	2 (14%)	2 (50%)
Posterior cerebral artery	2 (12%)	0 (0%)	2 (50%)
Surgical treatment, *n* (%)	4 (22%)	4 (28%)	0 (0%)
Endovascular treatment, *n* (%)	10 (56%)	8 (58%)	2 (50%)
History of hypertension, *n* (%)	10 (56%)	8 (58%)	2 (50%)
History of smoking, *n* (%)	5 (28%)	4 (28%)	1 (25%)
In-hospital mortality, *n* (%)	9 (50%)	6 (43%)	3 (75%)
APACHE II scale [points]	18 [15–22]	19 [15–22]	19 [18–20]
SAPS II scale [points]	44 [38–56]	43 [37–53]	61 [52–62]

SAH—Subarachnoid Hemorrhage, APACHE II—Acute Physiology And Chronic Health Evaluation II, SAPS II—Simplified Acute Physiology Score.

**Table 2:** Comparison of groups with cardiogenic and non-cardiogenic ARF etiology on ICU admission.

Variable	Non-Cardiogenic Phenotype *N* = 14	Cardiogenic Phenotype *N* = 4	*p*
Age [years]	64 [47–69]	44 [34–48]	0.08
Women, *n* (%)	8 (57%)	3 (75%)	0.64
GCS [points]	9 [5–14]	7 [5–10]	0.5
Fisher scale [points]	4 [2–4]	4 [2–4]	0.88
Hunt–Hess scale [points]	4 [2–4]	4 [4, 5]	0.19
WFNS scale [points]	4 [2–5]	4 [4, 5]	0.65
OI [mmHg]	296 [257–345]	190 [177–238]	**0.046**
Metanephrine [ng/mL]	63.5 [33–89]	249 [136–357]	**0.007**
IL-6 [pg/mL]	34 [22–64]	8.3 [4.6–10.3]	**0.013**
IL-1B [pg/mL]	0.37 [0.36–0.52]	0.8 [0.79–0.8]	0.09
Lactate [mmol/L]	2.1 [1.59–2.99]	3.19 [2.21–7.2]	0.32
CRP [mg/dL]	7.3 [2.5–19.6]	3.55 [1.4–8.25]	0.23
PCT [ng/mL]	0.18 [0.068–1.77]	0.54 [0.18–0.88]	0.79
LVEF [%]	50 [40–50]	37.5 [22.5–45]	0.23

GCS—Glasgow Coma Scale, OI—Oxygenation Index, IL-6—Interleukin 6, IL-1B—Interleukin 1B, CRP—C-Reactive Protein, PCT—Procalcitonin, LVEF—Left Ventricular Ejection Fraction.

## Data Availability

For legal reasons data are available from the authors of the study and can be made available upon request to other scientific institutions.
